# EHPnet: Stop TB Partnership

**Published:** 2008-11

**Authors:** Erin E. Dooley

http://www.stoptb.org/

In 2000 international agencies, government ministers, and donor groups formed the Stop TB Partnership with the mission of eliminating tuberculosis (TB). The partnership’s Global Plan to Stop TB 2006–2015, one of its central efforts, is a comprehensive survey of the steps and resources the Stop TB partners believe will be needed to make the greatest impact on the global TB burden. The Plan section of the Stop TB website features updates on progress toward meeting the plan’s goals and bridging a $30.8 billion funding gap.

The site also highlights the Global Drug Facility (GDF), established as a mechanism to expand implementation of “daily observed treatment, short course” programs by facilitating consistent access to high-quality TB drugs. In 2007, the GDF funded treatment for more than a million people in 44 countries.

The Working Groups section of the site describes the activities of seven groups coordinating various aspects of TB control. Among other areas, these groups address multidrug-resistant TB as well as new vaccines, treatments, and diagnostics. The Stop TB Resource Centre links to additional tools, including fact sheets, reports, guides, a TB glossary, and video and photo libraries.

